# Cancer risk in individuals with polydactyly: a Swedish population-based cohort study

**DOI:** 10.1038/s41416-024-02770-z

**Published:** 2024-06-29

**Authors:** Alexandra Wachtmeister, Giorgio Tettamanti, Ida Nordgren, Christina Norrby, Tobias Laurell, Yunxia Lu, Anna Skarin Nordenvall, Ann Nordgren

**Affiliations:** 1 Department of Molecular Medicine and Surgery, Karolinska Institutet, Stockholm, Sweden; 2 Unit of Epidemiology, Institute of Environmental Medicine, Karolinska Institutet, Stockholm, Sweden; 3 Department of Hand Surgery, Södersjukhuset, Stockholm, Sweden; 4 Department of Population Health and Disease Prevention & Department of Epidemiology and Biostatistics, Program in Public Health, University of California, Irvine, CA USA; 5 Department of Radiology, Karolinska University Hospital, Stockholm, Sweden; 6 Department of Clinical Genetics, Karolinska University Laboratory, Karolinska University Hospital, Stockholm, Sweden; 7 Department of Clinical Genetics and Genomics, Sahlgrenska University Hospital, Gothenburg, Sweden; 8 Institute of Biomedicine, Department of Laboratory Medicine, University of Gothenburg, Gothenburg, Sweden

**Keywords:** Paediatric cancer, Epidemiology, Risk factors, Cancer

## Abstract

**Background:**

Polydactyly is a feature of several cancer predisposition syndromes (CPS), however, cancer risk in individuals with polydactyly is largely unknown.

**Methods:**

We performed a matched cohort study using data from Swedish national registers. We included 6694 individuals with polydactyly, born in Sweden between 1970–2017. Polydactyly was categorised as thumb polydactyly, finger polydactyly, polydactyly+ (additional birth defects and/or intellectual disability) or isolated polydactyly. Each exposed individual was matched to 50 comparisons by sex, birth year and birth county. Associations were estimated through Cox proportional hazard models.

**Findings:**

An increased childhood cancer risk was found in males (HR 4.24, 95% CI 2.03–8.84) and females (HR 3.32, 95% CI 1.44–7.63) with polydactyly+. Isolated polydactyly was associated with cancer in childhood (HR 1.87, 95% CI 1.05–3.33) and young adulthood (HR 2.30, 95% CI 1.17–4.50) in males but not in females. The increased cancer risk remained after exclusion of two known CPS: Down syndrome and neurofibromatosis. The highest site-specific cancer risk was observed for kidney cancer and leukaemia.

**Conclusions:**

An increased cancer risk was found in individuals with polydactyly, especially in males and in individuals with polydactyly+. We encourage future research about polydactyly and cancer associations and emphasise the importance of clinical phenotyping.

## Introduction

Despite extensive research, the specific cause of most cancers remains unknown [1, 2]. Evidence suggests that cancer in children and young adults are strongly associated with germline pathogenic variants in genes that cause dysregulation of normal development [3–5]. To date, germline genetic aberrations are estimated to account for at least 10–15% of childhood cancers [6–8]. More than 60 cancer predisposition syndromes (CPS) and nearly 200 different genes have been described as strongly associated with cancer risk. The number of CPS is constantly growing, and recent studies suggest that genetic predisposition is much more common than previously thought [8–10]. In addition, increasing evidence suggests that individuals with birth defects stand an increased risk of developing cancer during childhood and adulthood [11–16]. The highest risk is reported for childhood cancer in individuals with both chromosomal aberrations and birth defects. However, individuals with non-chromosomal birth defects are also at increased risk of cancer, especially during childhood [12–15].

Approximately, 1 in every 30 children is born with a birth defect [17]. Polydactyly is among the most common congenital limb defects [17, 18]. Individuals with polydactyly have extra digital parts, e.g., supernumerary fingers or toes. The occurrence of polydactyly differs among populations, however, overall prevalence in Europe is ~1 in every 1000 live births [17]. The patient group is heterogeneous with large phenotypic variability, ranging from small nubbins to fully developed digits [18]. Most commonly, polydactyly appears as an isolated clinical feature, but it is also a common feature in multiple syndromes [19, 20]. Polydactyly is associated with over 300 well-categorised syndromes [19] whereof at least 13 are known to be associated with cancer (see Supplementary Table 1). The current praxis is to surgically remove the extra digit within the first years of life [21].

Polydactyly is currently regarded as an isolated event unless other combinations of congenital anomalies raise suspicion of an underlying syndrome. Consequently, after surgical removal of the extra digit, no further precautions are taken. Growing evidence shows that individuals with birth defects are at increased risk of cancer and there are several known cancer predisposition syndromes associated with polydactyly. Despite this, to the best of our knowledge, there are no previous studies investigating the specific association between polydactyly and cancer.

## Methods

The associations between polydactyly and cancer were investigated through a matched cohort design based on data from Swedish national administrative- and healthcare registers. Linkage between the registers is enabled by the unique personal identity number assigned to all Swedish residents [22]. The study was conducted in accordance with the Strengthening the Reporting of Observational Studies in Epidemiology reporting guidelines.

Data from the following registers held by the Swedish National Board of Health and Welfare was used: the Medical Birth Register (MBR), the National Patient Register (NPR) and the National Cancer Register (NCR). The MBR holds information on birth characteristics, congenital malformations and perinatal diagnoses for all children born in Sweden since 1973 [23]. The NPR contains information on inpatient visits at Swedish hospitals since 1964, with national coverage since 1987 and includes data on outpatient visits since 2001 [24]. Both the NPR and MBR record diagnoses according to the International Classification of Diseases (ICD). Since 1958 it has been compulsory in Sweden to report all malignant tumours, leukaemias and certain premalignant and benign tumours to the NCR together with the corresponding ICD/ICD-O-code. The NCR has a high overall completeness and quality [25]. Information on date of birth, death, migration, birth county, parental ages and parental education was obtained from the Multi-Generation Register [26], the Total Population Register [27] and the Longitudinal Integration Database for Health Insurance and Labour Market Studies (Swedish acronym, LISA; 1990 and onward), all held by Statistics Sweden.

### Study design and participants

From the NPR and the MBR, we identified all individuals diagnosed with polydactyly born in Sweden between January 1, 1970, and December 31, 2017 with available data on county of birth (missing information *n* = 352; 5%, Supplementary Fig 1). Each individual with polydactyly was matched to 50 comparisons by sex, birth year and county of birth, identified through the Total Population Register.

### Identification of individuals with polydactyly

Our exposed cohort comprised individuals who had received a diagnostic code for polydactyly between 1970 and 2017, based on codes from ICD-8 (1969–1986), ICD-9 (1987–1996) and ICD-10 (1997-) (Supplementary Table 3). Individuals were defined as exposed if diagnosed with polydactyly at one or more occasions (in either the MBR or NPR), at any age in their life. To minimise the risk of exposure misclassification a supplementary sensitivity analysis restricting the cohort to individuals diagnosed with polydactyly at 5 years or younger was conducted. This age limit aimed to exclude individuals with an incidental wrong diagnosis while including individuals with complex or severe coexisting conditions where polydactyly diagnosis might be delayed.

For further exploration we investigated cancer risk with regards to the localisation of the extra digit and additional birth defects (for detailed information on subgroups and inclusion criteria see Supplementary Tables 2, 3).

Localisation was studied through subgroups ‘thumb polydactyly’ or ‘finger polydactyly’. Thumb polydactyly included individuals with an extra digit I. Finger polydactyly included individuals with an extra digit II, III, IV or V. Individuals with a diagnostic code for both ‘thumb polydactyly’ and ‘finger polydactyly’ were excluded from subgroup analyses to prevent misclassification (*n* = 205).

Cancer in polydactyly was further investigated in relation to additional birth defects through subgroups ‘polydactyly+’ or ‘isolated polydactyly’. Polydactyly+ included individuals with polydactyly and at least one additional non-associated birth defect (defined as malformations not involving the limbs) and/or intellectual disability. Polydactyly+ served as a proxy for the presence of a congenital malformation syndrome. Isolated polydactyly included individuals with polydactyly and no other birth defects and/or intellectual disability. Consequently, individuals with additional birth defects in the extremities were not considered in our subgroup analysis. Lastly, we conducted a sensitivity analysis excluding the two most common cancer predisposition syndromes, Down syndrome and neurofibromatosis, from the polydactyly+ cohort.

### Identification of cancer cases

Data on cancer diagnosis was retrieved from the National Cancer Registry (NCR) and classified according to the topography and morphology coding used in the NCR. Cancer diagnosis before age 20 was considered as childhood cancer. In analyses regarding childhood cancer, additional classifications were done using the *International Classification of Childhood Cancer*, third edition (ICCC-3) [28]. Tumours in the NCR were classified as malignant if the morphological code indicated malignancy, with exception for all CNS tumours, leukaemias and Hodgkin lymphomas that always were considered malignant [29]. Tumours were not classified as malignant if the morphology code indicated tumour as cancer in situ, uncertain if benign/malignant or benign [29]. Only the first cancer event was included.

### Statistical analysis

Descriptive statistics were used to summarise the study participants’ characteristics at baseline. Associations between polydactyly and cancer were estimated with Cox proportional hazard models with attained age as the underlying timescale, presented as hazard ratios (HRs) together with 95% confidence intervals (CI). All individuals were followed from birth until death, emigration, first cancer diagnosis, or end of follow-up (2017-12-31). All analyses were adjusted for sex, birth year, county of birth (categorised as six regions), maternal and paternal age at birth of index (categorised as <20, 20–29, 30–34, 35–39, >39 years) and highest attained level of parental education (categorised as primary, secondary, postsecondary). Statistical analyses were performed only if there were at least 5 cancer cases among individuals with polydactyly. Analyses stratified by sex and age were performed to explore differences in cancer risk. Schoenfeld’s residuals were used to test the proportional hazard assumption. Non-proportionality was indicated by an asterisk in the tables in Results. SAS 9.4 software was used for data preparation and Stata 16.1 was used to perform statistical analyses.

## Results

### Baseline characteristics

By using data from the MBR and the NPR we identified 6694 individuals born in Sweden with a polydactyly diagnosis between 1970–2017. These individuals were matched by age, sex and county of birth to 334,700 individuals without a polydactyly diagnosis: characteristics of the study participants are shown in Table 1. Of the study participants 57.3% were male and 42.8% were female. Nearly 14% of the individuals with polydactyly had a duplicated thumb (preaxial polydactyly, *n* = 926) while 38.1% (*n* = 2561) had a duplication of digit II–V. For the remaining cases (44.8%) the localisation was unspecified. Out of the total 6694 individuals with polydactyly, 4045 (60.4%) cases were isolated and 1694 (25.3%) had an additional non-associated birth defect and/or intellectual disability (polydactyly+).

**Table 1:** Characteristics of the study population.

	Polydactyly	Matched comparisons
	*No. (%)*	*No. (%)*
Total number of individuals	6694	334,700
Subgroups of polydactyly		
Thumb	926 (13.8%)	-
Finger^a^	2561 (38.2%)	-
Polydactyly+^b^	1694 (25.3%)	-
Isolated	4045 (60.4%)	-
Birth year		
1970–1979	867 (13.0%)	43,350 (13.0%)
1980–1989	1317 (19.7%)	65,850 (19.7%)
1990–1999	1333 (19.9%)	66,650 (19.9%)
2000–2009	1590 (23.8%)	79,500 (23.8%)
2010–2017	1587 (23.7%)	79,350 (23.7%)
Sex		
Male	3832 (57.3%)	191,600 (57.3%)
Female	2862 (42.8%)	143,100 (42.8%)
Maternal age at birth of index		
<20	122 (1.8%)	6229 (1.9%)
20–29	3372 (50.4%)	164,980 (49.3%)
30–34	1957 (29.2%)	102,955 (30.8%)
35–39	993 (14.8%)	48,955 (14.6%)
≥40	239 (3.6%)	11,170 (3.3%)
Missing data	11 (0.2%)	411 (0.1%)
* Mean age (SD)*	29.4 (±5.4)	29.5 (±5.3)
Paternal age at birth of index		
<20	40 (0.6%)	1303 (0.4%)
20–29	2271 (33.9%)	112,234 (33.5%)
30–34	2046 (30.6%)	107,965 (32.3%)
35–39	1325 (19.8%)	68,527 (20.5%)
≥40	897 (13.4%)	40,524 (12.1%)
Missing data	115 (1.7%)	4147 (1.2%)
* Mean age (SD)*	32.5 (±6.5)	32.4 (±6.2)
Highest parental education		
Primary	368 (5.5%)	16,392 (4.9%)
Secondary	2797 (41.8%)	134,461 (40.2%)
Postsecondary	3479 (52.0%)	181,394 (54.2%)
Missing data	50 (0.8%)	2453 (0.7%)
Age at end of follow-up		
<10	2211 (33.0%)	105,800 (32.6%)
10–19	1463 (21.9%)	73,983 (22.1%)
20–29	1377 (20.6%)	70,803 (21.2%)
30–39	1094 (16.3%)	56,460 (16.9%)
≥40	549 (8.2%)	27,617 (8.3%)
Cancer diagnosis		
All neoplasms	124 (1.9%)	5487 (1.6%)
* Median age at diagnosis (IQR)*	25.8 (12.8)	27.7 (9.3)
Malignant neoplasms	55 (0.8%)	2035 (0.6%)
* Median age at diagnosis (IQR)*	18.0 (25.8)	24.4 (24.0)
Other reasons for end of follow-up		
Emigration	443 (6.6%)	23,222 (6.9%)
* Median age at emigration (IQR)*	11.2 (20.0)	11.4 (21.1)
Death	203 (3.0%)	3438 (1.0%)
* Median age at death (IQR)*	0.3 (13.6)	4.1 (23.6)

Individuals with polydactyly diagnosed between 1970–2017 in Sweden. Age is defined in years.

^a^Finger polydactyly include extra digit 2, 3, 4 and 5.

^b^Including individuals with polydactyly and an additional birth defect (not in the extremities) and/or intellectual disability.

The study population was followed for a total of 6,615,369 person-years. For individuals with polydactyly, the median age at end of follow-up was 17.8 (IQR 22.8*)* years and the maximum age was 47 years. During follow-up, 203 (3.0%) individuals with polydactyly died, whereas 3438 (1.0%) of matched comparisons died (Table 1).

### Cancer risk in individuals with polydactyly

In total, 120 individuals with polydactyly had a cancer diagnosis and the median age at cancer diagnosis was 25.8 (IQR 12.8) years, see Table 1. The overall risk of cancer in polydactyly was borderline significant (HR 1.15, 95% CI 0.96–1.38). When stratifying by age, there was an increased risk of childhood cancer (HR 1.80, 95% CI 1.26–2.57). When further stratifying by sex, male individuals with polydactyly displayed a significantly increased risk of cancer, specifically for childhood cancer (HR 2.20, 95% CI 1.42–3.41) and cancer at 20–29 years of age (HR 2.17, 95% CI 1.24–3.79) (Table 2). There was no association between polydactyly and cancer in females in the main analysis.

**Table 2:** Cancer risk in individuals with polydactyly and subgroups of polydactyly born in Sweden between 1970–2017, stratified by age and sex.

	Polydactyly		Isolated polydactyly		Polydactyly+^a^		Polydactyly+ excl. NF & DS^b^	
	No. cases polydactyly/comparisons	aHR^c^ (95% CI)	No. cases polydactyly/comparisons	aHR^c^ (95% CI)	No. cases polydactyly/comparisons	aHR^c^ (95% CI)	No. cases polydactyly/comparisons	aHR^c^ (95% CI)
All neoplasms	120/5286	1.15 (0.96–1.38)^d^	75/3414	1.10 (0.87–1.38)	30/1187	1.34 (0.93–1.93)^d^	26/1182	1.21 (0.82–1.78)
*Female*	79/4159	0.96 (0.76–1.20)	52/2645	0.98 (0.74–1.30)^d^	16/959	0.88 (0.53–1.44)^d^	15/957	0.85 (0.51–1.42)
*Male*	41/1127	1.89 (1.38–2.58)	23/769	1.52 (1.01–2.31)^d^	14/228	3.51 (2.03–6.06)^d^	11/225	2.84 (1.54–5.25)
Malignant neoplasms	55/2035	1.40 (1.07–1.83)	29/1338	1.12 (0.77–1.62)	23/447	2.76 (1.81–4.21)	19/442	2.37 (1.49–3.77)
*Female*	20/1032	1.00 (0.64–1.56)	10/656	0.80 (0.43–1.50)	9/244	1.92 (0.98–3.75)	8/242	1.77 (0.87–3.59)
*Male*	35/1003	1.80 (1.28–2.53)	19/682	1.42 (0.90–2.25)	14/203	3.89 (2.24–6.75)	11/200	3.17 (1.71–5.87)
Age at diagnosis								
0–19	31/893	1.80 (1.26–2.57)	15/572	1.35 (0.81–2.25)	14/206	3.65 (2.11–6.32)	12/203	3.25 (1.80–5.85)
*Female*	10/397	1.32 (0.70–2.47)^d^	<5/244	n/a	6/99	3.32 (1.44–7.63)	6/105	3.34 (1.35–7.68)
*Male*	21/496	2.20 (1.42–3.41)^d^	≥5/328	1.87 (1.05–3.33)^d^	8/107	4.24 (2.03–8.84)	6/98	3.28 (1.42–7.61)
20–29	54/2446	1.13 (0.86–1.48)	33/1505	1.11 (0.78–1.57)	9/582	0.81 (0.42–1.57)	8/582	0.74 (0.37–1.49)
*Female*	41/2139	0.98 (0.72–1.34)	24/1308	0.93 (0.62–1.39)	≥5/509	0.82 (0.41–1.65)	≥5/509	0.73 (0.35–1.55)
*Male*	13/307	2.17 (1.24–3.79)	9/197	2.30 (1.17–4.50)	<5/73	n/a	<5/73	n/a
30 and older	35/1947	0.88 (0.63–1.24)	27/1402	0.98 (0.67–1.45)	7/399	0.94 (0.45– 2.00)	6/397	0.85 (0.34–1.91)
*Female*	28/1623	0.83 (0.57–1.22)	≥5/1093	1.11 (0.74–1.67)	<5/351	n/a	<5/350	n/a
*Male*	7/324	1.13 (0.53–2.41)	<5/244	n/a	≥5/48	7.57 (2.85–20.07)	<5/47	n/a

Presented are adjusted hazard ratios (aHRs) together with 95% confidence intervals (CI).

^a^Individuals with polydactyly and additional non-associated birth defects and/or intellectual disability.

^b^Individuals in polydactyly+ cohort excluding individuals with Down Syndrome (DS) or neurofibromatosis (NF).

^c^Adjusted for sex, county of birth, paternal age, maternal age and parental education.

^d^Not meeting proportional hazards assumption.

### Cancer risk in subgroup analyses

In subgroup analyses, individuals with polydactyly+ had an overall increased risk of malignant neoplasms (HR 2.76, 95% CI 1.81–4.21, Table 2). Age-stratified analyses displayed an increased risk of childhood cancer (HR 3.65, 95% CI 2.11–6.32). The association was consistent in both male and female individuals (HR 4.24, 95% CI 2.03–8.84 resp. HR 3.32, 95% CI 1.44–7.63). In addition, males with polydactyly+ had a significantly increased risk for cancer >30 years (HR 7.57, 95% CI 2.85–20.07), however, the analysis was based on few co-occurring cases. The cancer risk seen throughout analyses for polydactyly+ was slightly reduced, but remained significantly increased, when excluding subjects with Down syndrome or neurofibromatosis from the polydactyly+ cohort (Table 2).

In individuals with polydactyly+ and cancer, 12 out of 30 individuals were diagnosed with a syndrome and six individuals had intellectual disability (Table 3). The following groups of syndromes were represented; neurocutaneous syndromes (*n* = 3), chromosomal abnormalities (*n* = 3), overgrowth syndromes (*n* = 2) and the remaining had other specified congenital malformation syndromes (*n* = 4). The most common additional malformations for individuals with polydactyly+ were musculoskeletal, malformations of the genital organs, and intellectual disability (Table 3).

**Table 3:** Specification of birth defects in the group of 30 individuals with ‘Polydactyly+’ and cancer.

Site of malformation	Frequency of malformation
Skeletal and muscles	6
Genital organs	6
Intellectual disability	6
Ear	4
Circulatory system	4
Skin	4
Nervous system	3
Urinary organs	3
Other malformations	7
Total number of individuals diagnosed with a specific syndrome	12

Birth defects (BDs) in the 30 individuals with polydactyly+ and cancer, diagnosed between 1970–2017 in Sweden. In addition to polydactyly each individual have one or more BD and/or intellectual disability. The BDs are presented and classified in regard to localisation or organ system affected.

Among individuals with isolated polydactyly, an increased cancer risk was observed only in males aged 0–19 and 20–29 (HR 1.87, 95% CI 1.05–3.33, HR 2.30, 95% CI 1.17–4.50, respectively). No increased risk was found in women with isolated polydactyly (Table 2).

There were no associations when analysing cancer risk with regard to localisation of the duplicated digit (thumb polydactyly: HR 1.07, 95% CI 0.68–1.70 and finger polydactyly: HR 0.94, 95% CI 0.66–1.33) (Supplementary Table 4).

### Site-specific cancer risk

In analyses on site-specific cancer, an increased risk of cancer was found for leukaemia and kidney cancer (Table 4). There was a two-fold increase in risk of leukaemia observed in individuals with polydactyly, mainly driven by acute lymphoblastic leukaemia (ALL) (HR 3.38, 95% CI 1.36–8.41). The highest site-specific cancer risk was observed for kidney cancer, in which all five kidney cancer cases were male (HR of 7.87, 95% CI 2.94–21.09) (Table 4).

**Table 4:** Risk of site-specific cancer for individuals born with polydactyly in Sweden between 1970–2017, in general and stratified by sex.

Site of cancer	All polydactyly		Male		Female	
	No. cases polydactyly/comparisons	aHR^a^ (95% CI)	No. cases polydactyly/comparisons	aHR^b^ (95% CI)	No. cases polydactyly/comparisons	aHR^b^ (95% CI)
Leukaemia	11/275	2.10 (1.15–3.84)	≥5/158	3.04 (1.54–5.98)	<5/117	n/a
ALL	5/124	2.11 (0.86–5.18)	5/78	3.38 (1.36–8.41)	−/46	-
Lymphoma	5/219	1.18 (0.49-2.88)	<5/129	n/a	>5/90	n/a
Kidney	5/50	5.26 (2.06–13.44)	5/32	7.87 (2.94–21.09)	−/18	-
CNS and meningies	10/332	1.62 (0.86–3.05)	<5/192	n/a	≥5/140	2.30 (1.01–5.24)
Gynaecological	58/2988	0.97 (0.75–1.26)	-	-	58/2988	0.97 (0.75–1.26)
Cervix	54/2899	0.93 (0.71–1.22)	-	-	54/2899	0.93 (0.71–1.22)
Testis	5/199	1.27 (0.52–3.10)	5/199	1.27 (0.52–3.10)	-	-
Melanoma	9/358	1.30 (0.67–2.52)	≥5/130	1.99 (0.81–4.89)	<5/228	n/a
Endocrine	6/219	1.47 (0.65–3.32)	<5/63	n/a	<5/156	n/a

Presented are adjusted hazard ratios (aHRs) together with 95% confidence intervals (CI). Gynaecological site includes cervix, ovary, uterus, vulva and vaginal cancer.

*ALL* acute lymphoblastic leukaemia.

^a^Adjusted for sex, county of birth, paternal age, maternal age and parental education.

^b^Adjusted for county of birth, paternal age, maternal age and parental education.

There was a high prevalence of cervical neoplasms in the female cohort, explained by a high frequency of pre-cancerous high-grade squamous intraepithelial lesions (HSIL) and high-grade dysplasia identified through the Swedish national cervical screening programmes. The risk of cervical neoplasms was not increased in women with polydactyly.

## Discussion

In this registry-based cohort study, including 6 694 individuals with polydactyly born between 1970 and 2017 in Sweden, we found that individuals with polydactyly had a significantly increased risk of cancer early in life, especially in males. The highest risk estimates were observed in male individuals with polydactyly+ (with a four-fold increased risk of childhood cancer and seven-fold increased risk of cancer over 30 years). The increased risk remained even after exclusion of the two known cancer predisposing syndromes; neurofibromatosis and Down syndrome. Notably, also male individuals with isolated polydactyly had an increased risk of cancer in early life. The association between polydactyly+ and childhood cancer was the only significant association seen in both male and female subjects. Beyond that, female subjects with polydactyly did not have an increased risk of cancer. In analyses on site-specific cancer risk the highest risk was observed for leukaemia, especially ALL and kidney cancer, in males with polydactyly.

Albeit increased, the absolute childhood cancer risk in individuals with polydactyly remains low. The findings of our study indicate an absolute childhood cancer risk of ~0.5% for male individuals with polydactyly and 0.8% for male individuals with polydactyly+.

In all our analyses, male individuals with polydactyly were at a higher risk of developing cancer than female individuals. The risk of cancer in male subjects was increased for individuals with polydactyly in general, for individuals with polydactyly+ and for individuals with isolated polydactyly. It is well-known that differences in sex affect cancer incidence and mortality [30] and for most cancer types, the incidence is generally higher for males during childhood and adolescence as compared to females [31]. X-linked disorders, differences in gene expression on X-and autosomal chromosomes, immune response and hormonal activity are likely to play a role in cancer susceptibility in males [31–33]. In addition, male individuals have a higher incidence of birth defects [34]. Despite this, only two recent studies have considered sex differences when evaluating cancer risk in for individuals with birth defects [35, 36]. Marcotte et al. propose that birth defects act as a strong mediator between sex and childhood cancer, explaining up to 40% of the association [36]. In contrast, Daltveit et al. suggest only a small (4.8%) mediating effect of birth defects for the association between sex and paediatric cancers and a higher cancer risk in females than males with birth defects [35]. Nonetheless, as our comparisons were matched by sex, our results indicate that there is a true association between polydactyly and cancer, not only mediated by a sex-cancer association. Furthermore, our results suggest that a shared unknown factor in male subjects makes them more prone to develop cancer if they have polydactyly.

The strongest associations in our study were found between male subjects with polydactyly+ and cancer. As polydactyly+ is likely to represent a syndromic population with a large proportion harbouring genetic aberrations [37], our results could suggest that male individuals with polydactyly (in contrast to female individuals with polydactyly) are more likely to have a syndrome that predisposes for cancer and/or that male individuals with polydactyly are more vulnerable to develop cancer if having a cancer predisposition syndrome. Known monogenic X-linked syndromes with associations to polydactyly and cancer, such as Simpson-Golabi-Behmel Syndrome (presented in Supplementary Table 1), could partly explain the male cancer risk in our study. It is however likely that the underlying causes are heterogenous and that there are hitherto undescribed disease-causing genes and mechanisms behind polydactyly and cancer development.

Since polydactyly+ represents polydactyly with additional birth defects and/or intellectual disability, our findings are in accordance with previous studies suggesting a positive correlation between the risk of childhood cancer and an increasing number of birth defects [11, 13, 14]. In individuals with isolated polydactyly, only a slightly increased risk for cancer among males with polydactyly aged 0–19 and 20–29 was observed. Consequently, the cancer risk found in our study could mainly, although not fully, be attributed to the subgroup of polydactyly+.

Having multiple birth defects and/or intellectual disability raises the suspicion of an underlying genetic syndrome. As anticipated, a large proportion (12 out of 32) of the individuals with polydactyly+ and cancer had a diagnostic code for a specific syndrome. The most common syndromes were chromosomal disorders, neurocutaneous syndromes and overgrowth syndromes, which correlate well to the most common CPS; Down syndrome and Neurofibromatosis 1 [38]. Overgrowth syndromes (such as Beckwith-Wiedemann) also have well-established cancer risk [39]. Polydactyly has previously been described in all the aforementioned syndromes although as an uncommon part of the phenotype (Supplementary Table 1) [40–42]. When removing all individuals with neurofibromatosis and Down syndrome from our cohort, individuals with polydactyly+ had a slightly lower, although still increased, risk of cancer. Hence, our results support the increased cancer risk previously described in these CPS, but the risk for individuals with polydactyly+ cannot solely be explained by Down syndrome and neurofibromatosis.

The most common birth defects in individuals with polydactyly+ were those of the muscles and skeleton, genital organs, intellectual disability and/or ears (Table 3) which somewhat differs from the most common birth defects in the general population; cardiac malformations, intellectual disability, genital anomalies and oro-facial clefts [43–45]. As the distribution of birth defects differs from the general population, our results could indicate that there are hitherto undiscovered CPS that contribute to the increased risk of cancer for individuals with polydactyly and/or that polydactyly may be an unrecognised expanded phenotype in already known congenital CPS.

In our site-specific cancer analysis, the highest cancer risk was found for leukaemia and kidney cancer. Male subjects had an 8-fold increased risk of kidney cancer. Several overgrowth syndromes report an increased risk for embryonal tumours such as Wilms tumour, hepatoblastoma, neuroblastoma and others [46]. Beckwith-Wiedemann and Simpson-Golabi-Behmel Syndrome are two well-known overgrowth syndromes with risk of embryonal tumours and are also association to polydactyly [46] (Supplementary Table 1). These syndromes may contribute to the increased risk of kidney cancer observed in our study. For leukaemia predisposition, children and young adults with genetic syndromes, e.g. bone marrow failure syndromes and telomeropathies, have an increased predisposition to developing haematological malignancies and it is possible that some cases could be explained by known leukaemia-associated syndromes such as Down syndrome, Fanconi Anemia and Diamond-Blackfan Anemia, which are also associated with polydactyly [4].

As the increased cancer risk in male individuals remain significant in both isolated polydactyly and polydactyly+ the X-linked CPS described above are unlikely to solely explain the male predisposition shown in this study. The mechanisms behind how and why polydactyly, and potentially other birth defects, are associated with increased male cancer susceptibility remains largely unknown.

## Strengths and limitations

The main strength of this study is the use of high-quality Swedish registry data with wide coverage, detailed information and high validity. The large sample size enables sex- and age stratification, and subgroup analysis, through which we were able to reveal high-risk populations within the cohort. Without these sub-analyses significant associations would be missed.

Due to the rarity of polydactyly and cancer in children and young adults, a limitation to this study, and a challenge for studies of childhood cancer in general, is the limited number of cancer cases. Consequently, we did not have enough cases to evaluate rare subgroups of childhood cancers such as Wilms tumour, hepatoblastoma and medulloblastoma separately. Identification of polydactyly was based solely on register data and could not be verified by us in the clinic or by review of medical records, increasing the risk of misclassification of exposure. Disparities in registering in the NPR and medical records have been reported for congenital upper limb anomalies. However, these disparities have been speculated to consist mainly of the more subtle anomalies appearing later in life such as camptodactyly and congenital trigger digits [47]. Since polydactyly is visible at birth and easy to diagnose, misclassification is presumably a minor source of bias. To test the robustness of our results, we performed a sensitivity analysis, including only individuals who received a polydactyly diagnosis at 5 years of age or younger. The results from the sensitivity analysis supports the findings in the full cohort (Supplementary Tables 5, 6).

Lastly, as the NPR does not reach nationwide coverage until 1987 and polydactyly is often surgically corrected in young age, patients with polydactyly born in the beginning of our study may lack a diagnosis in the register and thus have been included as comparisons. However, given the rarity of polydactyly, we estimate this risk as low and addressed this limitation by matching on birth year and birth county in order to make detection bias non-differential.

### Clinical relevance & future implications

Approximately 1–2 out of 10,000 children and 5–10 out of 10,000 young adults (age 20–39) develop cancer worldwide [48, 49]. Additionally, 3% of all individuals are born with a congenital anomaly [17]. Birth defects are one of the few known risk factors for childhood cancer, however, the extent of the risk is still not known for most birth defects. Identifying particular groups of individuals who are at high risk of cancer enables effective surveillance and screening strategies, and also elucidates topics suitable for future research. Generally, cancer surveillance programmes are considered appropriate for conditions with >5% risk of developing childhood cancer. However, this threshold is debatable and for conditions with 1–5% cancer risk, an individual risk-benefit assessment is often appropriate [50]. There is no equivalent threshold for adults, and recommendations are tailored based on comprehensive condition-specific assessments [51]. In our study, the absolute childhood cancer risk for male individuals with polydactyly (0.5%) and polydactyly+ (0.8%) does not meet the suggested threshold for childhood cancer surveillance.

An increasing number of syndromes and birth defects have been associated with cancer predisposition in the last decades [1, 9]. As polydactyly may be part of the clinical manifestation of cancer predisposition syndromes our results reinforce the value of careful clinical phenotyping in patients with cancer. It is of great importance to diagnose cancer predisposition syndromes since this may result in tailored surveillance or altered treatment recommendations. To evaluate all aspects of birth defects’ association with cancer, we therefore encourage future epidemiological and genetic studies to investigate birth defects both in the context of syndromes (representing known and unknown CPS) and as isolated features, in both children and adults. Furthermore, this study highlights the importance of sex differences in cancer predisposition and encourages future studies to take this correlation into close consideration.

## Conclusion

This large cohort study on individuals with polydactyly presents an increased cancer risk during childhood and young adulthood, predominately in male subjects. The risk was especially elevated in individuals with additional birth defects and/or intellectual disability. We hypothesise that the increased risk could be due to known and hitherto undiscovered CPS and reinforce the value of careful clinical phenotyping in patients with cancer and/or polydactyly. Furthermore, we encourage future researchers to explore the extent of the male predisposition seen in this study and to closely consider sex differences when evaluating birth defects’ association to cancer.

### Supplementary information

Supplementary information

## Data Availability

The datasets analysed in this study are not publicly available according to Swedish laws and regulations to protect participants confidentiality. Sensitive personal data can only be made available for researchers who fulfil legal requirements. Data are located in controlled access data storage at Karolinska Institutet.
