# Supplementary material for: Cancer risk in individuals with polydactyly: a Swedish population-based cohort study
Source: Br J Cancer. 2024 Jun 29;131(4):755–62. doi: 10.1038/s41416-024-02770-z (PMC11333495; doi:10.1038/s41416-024-02770-z)
Supplement: Supplementary file 1 — Supplementary information [file 41416_2024_2770_MOESM1_ESM.pdf]

Supplementary information

Supplementary Figure 1. Cohort flow chart.
Supplementary Table 1. Examples of syndromes associated with both polydactyly and cancer.
Supplementary Table 2. Inclusion criteria for subgroups of polydactyly. Description of individuals included in each subgroup.
Supplementary Table 3. List of ICD-codes used for inclusion criteria. ICD-codes for polydactyly and subgroups of polydactyly.
Supplementary Table 4. Cancer risk in regard to localization of the duplicated digit. Presented as hazard ratio and 95% confidence intervals.
Supplementary Table 5. Sensitivity analysis of cancer risk in individuals diagnosed with polydactyly at 5 years of age or younger. Risk is presented as hazard ratio together with 95% confidence intervals.
Supplementary Table 6. Sensitivity analysis of site-specific cancer in individuals diagnosed with polydactyly at 5 years of age or younger. Risk is presented as hazard ratio together with 95% confidence intervals.

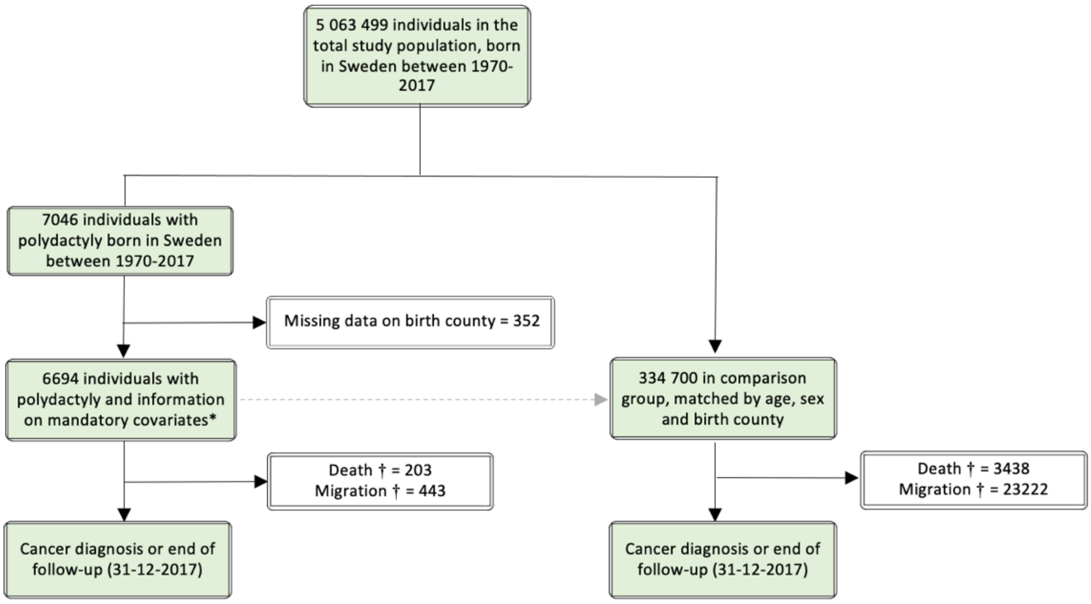

Supplementary Figure 1. Cohort flow chart.
* Sex, birth year and county of birth
† Prior to cancer diagnosis

Supplementary Table 1. Examples of syndromes associated with both polydactyly and cancer, identified through Mendelian Inheritance in Man (OMIM-database) and PubMed.

Name of syndrome	OMIM-number	Gene	Inheritence	Associated cancers
Basal Cell Nevus Syndrome (Gorlin-Goltz)	#109400	PTCH1, PTCH2, SUFU	AD	Basal cell carcinoma, medulloblastoma[1]
Beckwith-Wiedemann Syndrome	#130650	ICR1, KCNQ1OT1, CDKN1C	AD	Embryonal tumors[2]
Bloom Syndrome	#210900	RECQL3	AR	Leukemia, lymphoma, various solid tumors[1]
Curry Jones Syndrome	*#601707*	SMO	SMu	Medulloblastoma[3,4]
Diamond-Blackfan Anemia	#105650	RPS19	AD	Leukemia (especially AML), various solid tumors[5]
Down Syndrome	#190685	Trisomy 21	-	Leukemia (especially AML)[6]
Fanconi Anemia	#227650, and more	FANC-genes, BRCA-genes, etc	AR, XLR	Leukemia (especially AML), various solid tumors[7]
MCAP†	#602501	PIK3CA	SMu	Wilms tumor, meningioma[1]
Neurofibromatosis 1	#162200	NF1	AD	CNS tumors, nerve sheath tumors and others[8,9]
Rubinstein-Taybi Syndrome	#180849	CREBBP	AD	Various tumors (especially of the head)[1]
Schinzel-Giedion Midface Retraction Syndrome	#269150	SETBP1	AD	Embryonal tumors[10]
Simpson-Golabi-Behmel Syndrome	#312870	GPC3	XLR	Embryonal tumors[11]
WAGR*	#194072	WT1, PAX6	AD, SMu	Wilms tumor[12,13]

Syndromes are presented together with OMIM-number, associated gene, inheritance pattern and most commonly associated cancer type. AD=autosomal dominant, AR=autosomal recessive, XLR=X-linked recessive, Smu=somatic mutation.
† = Megalencephaly-Capillary Malformation-Polymicrogyria Syndrome
* = Wilms Tumor, Aniridia, Genitourinary Anomalies, And Mental Retardation Syndrome

[1] Online Mendelian Inheritance in Man (OMIM) [Internet]. McKusick-Nathans Institute of Genetic Medicine. [cited 2023 Oct 2]. Available from: Available from: http://www.omim.org/

[2] Brioude F, Kalish JM, Mussa A, Foster AC, Bliek J, Ferrero GB, et al. Expert consensus document: Clinical and molecular diagnosis, screening and management of Beckwith-Wiedemann syndrome: an international consensus statement. Nat Rev Endocrinol. 2018;14(4):229-49.

[3] Porath B, Farooki S, Gener M, Amudhavalli SM, Grote L, Cooley LD, et al. Occurrence and characterization of medulloblastoma in a patient with Curry-Jones syndrome. Clin Genet. 2020;97(4):670-1.

[4] Grange DK, Clericuzio CL, Bayliss SJ, Berk DR, Heideman RL, Higginson JK, et al. Two new patients with Curry-Jones syndrome with trichoblastoma and medulloblastoma suggest an etiologic role of the sonic hedgehog-patched-GLI pathway. Am J Med Genet A. 2008;146a(20):2589-97.

[5] Vlachos A, Ball S, Dahl N, Alter BP, Sheth S, Ramenghi U, et al. Diagnosing and treating Diamond Blackfan anaemia: results of an international clinical consensus conference. Br J Haematol. 2008;142(6):859-76.

[6] Hasle H, Clemmensen IH, Mikkelsen M. Risks of leukaemia and solid tumours in individuals with Down's syndrome. Lancet. 2000;355(9199):165-9.

[7] Alter BP. Cancer in Fanconi anemia, 1927-2001. Cancer. 2003;97(2):425-40.

[8] Leppavirta J, Kallionpaa RA, Uusitalo E, Vahlberg T, Poyhonen M, Peltonen J, et al. Congenital anomalies in neurofibromatosis 1: a retrospective register-based total population study. Orphanet J Rare Dis. 2018;13:8.

[9] Uusitalo E, Rantanen M, Kallionpää RA, Pöyhönen M, Leppävirta J, Ylä-Outinen H, et al. Distinctive Cancer Associations in Patients With Neurofibromatosis Type 1. J Clin Oncol. 2016;34(17):1978-86.

[10] Lehman AM, McFadden D, Pugash D, Sangha K, Gibson WT, Patel MS. Schinzel-Giedion syndrome: report of splenopancreatic fusion and proposed diagnostic criteria. Am J Med Genet A. 2008;146a(10):1299-306.

[11] Sajorda BJ, Gonzalez-Gandolfi CX, Hathaway ER, Kalish JM. Simpson-Golabi-Behmel Syndrome Type 1. In: Adam MP, Everman DB, Mirzaa GM, Pagon RA, Wallace SE, Bean LJH, et al., editors. GeneReviews(®). Seattle (WA): University of Washington, Seattle

[12] Manoukian S, Crolla JA, Mammoliti PM, Testi MA, Zanini R, Carpanelli ML, et al. Bilateral preaxial polydactyly in a WAGR syndrome patient. Am J Med Genet A. 2005;134(4):426-9.

[13] Bremond-Gignac D, Gerard-Blanluet M, Copin H, Bitoun P, Baumann C, Crolla JA, et al. Three patients with hallucal polydactyly and WAGR syndrome, including discordant expression of Wilms tumor in MZ twins. Am J Med Genet A. 2005;134(4):422-5.

Supplementary Table 2. Description and inclusion criteria for subgroups of polydactyly.

Group name	Description of group	Include individuals with ICD-codes from:
Polydactyly	All individuals with polydactyly	Group A (S3 Table)
Thumb polydactyly*	Individuals with an additional thumb or triphalangeal thumb	Group B (S3 Table)
Finger polydactyly†	Individuals with an additional finger (digit II-V)	Group C (S3 Table)
Polydactyly+	Individuals with polydactyly and an additional birth defect not involving the extremities and/or intellectual disability	Group A and group D and/or group E (S3 Table)
Polydactyly+ excluding DS & NF	Polydactyly+ according to explaination above, excluding individuals with Down syndrome or Neurofibromatosis	According to above, excluding group F (S3 Table)
Isolated polydactyly	Individuals with polydactyly without any additional birth defects or intellectual disability	Group A, exclude all individuals in group E or group G (S3 Table)

* Could also be referred to as individuals with preaxial polydactyly

† Could also be referred to as individuals with central polydactyly and/or postaxial polydactyly

Supplementary Table 3. List of ICD-codes used for inclusion criteria. ICD-codes for polydactyly and subgroups of polydactyly.

Group	ICD-system	ICD-code	Code description
**A.** ICD-codes for polydactyly			
	ICD-8	755.00	Polydactyly, thumb
		755.01	Polydactylia digiti I, foot
		755.02	Polydactylia, finger V
		755.03	Polydactylia digiti V, foot
		755.04	Polydactylia other or unspecified, hand
		755.05	Polydactylia other or unspecified, foot
		755.06	Triphalangeal thumb
	ICD-9	755A	Polydactyly, accessory fingers or toes
	ICD-10	Q69	Polydactyly
		Q70.4	Polysyndactyly
**B.** ICD-codes for thumb polydactyly			
	ICD-8	755.00	Polydactyly, thumb
		755.06	Triphalangeal thumb
	ICD-9	NOT AVAILABLE	
	ICD-10	Q69.1	Accessory thumb
**C.** ICD-codes for finger polydactyly			
	ICD-8	755.02	Polydactylia, finger 5 (little finger)
		755.04	Polydactylia other or unspecified
	ICD-9	NOT AVAILABLE	
	ICD-10	Q69.0	Accessory finger
**D.** ICD-codes for congenital malformations that are not involving the arm, hand, leg or foot			
	ICD-8	740	Anencephaly
		741	Spina bifida
		742	Hydrocephalus
		743	Other malformations of the nervous system
		744	Eye malformation (not blindness)
		745	Congenital malformation of ear, face and neck
		746	Congenital heart malformations
		747	Other congenital malformation in circulatory system
		748	Congenital malformation in respiratory organs
		749	Cleft palate, lip and chin
		750	Congenital malformation in upper digestion system
		751	Other congenital malformation in digestion system
		752	Congenital malformation in genital organs
		753	Congenital malformations in urinary organs
		755.51	Cleidocranial dysostosis (CCD)
		755,6	Congenital hip dislocation (dislocatio congenita coxae (fixata))
		755.80	Arthrogryphosis multiplex congenita (several joint contractures)
		756	Other specified malformation in skeletal and muscles
		757	Congenital malformations of skin, hair and nails
		758	Other unspecified congenital malformations
		759	Congenital malformations in several organsystems
	ICD-9	740	Anencephali
		741	Spina bifida
		742	Other congenital malformations in the nervous system
		743	Congenital malformations of eye

	744	Congenital malformations of ear, face and neck
	745 - 746	Congenital heart malformation
	747	Other congenital malformation in the circulatory system
	748	Congenital malformation of circulatory system
	749	Cleft palate and lip
	750	Other congenital malformation in upper digestion system
	751	Congenital malformation in digestion system
	752	Congenital malformation of genital organs
	753	Congenital malformation of urinary organs
	754A-754C	Certain congenital deformations of face, skull, jaw, neck, spine
	754D	Congenital hip dislocation
	756	Other specified congenital malformations in muscles and skeleton
	757	Congenital malformation of skin, hair, nails and breastgland
	758	Chromosomal abberations
	759	Other and unspecified congenital malformations and syndromes
ICD-10	Q00-Q07	Congenital malformation of nervous system
	Q10-Q18	Congenital malformations of eye, ear, face and neck
	Q20-Q28	Congenital malformation of circulatory system
	Q30-Q34	Congenital malformations of respiratory organs
	Q35-Q37	Cleft palate and lip
	Q38-Q45	Other congenital malformations in digestion system
	Q50-Q56	Congenital malformations of genital organs
	Q60-Q64	Congenital malformations of urinary organs
	Q65	Congenital malformation in hip
	Q67	Congenital malformations of skull, face, spine or chest
	Q68.0	Congenital malgormation of sternocleidomastoid muscle
	Q74.3	Arthrogryposis multiplex congenita
	Q75-Q79	Other specified congenital malformation of skeleton and muscles
	Q80-Q89	Other specified congenital malformations and syndromes
	Q90-Q99	Chromosomal abberations that have not been classified anywhere else
	D82.1	Di Georges syndrom
	F84.2	Rett syndrome

E. ICD-codes for intellectual disability

ICD-8	310	Intell. lev. subnorm. ("borderline")
	311	Retardatio mentalis levis
	312	Retardatio mentalis moderata
	313	Retardatio mentalis gravis
	314	Retard. ment. prof. (idiotia)
	315	Retardatio mentalis nud
ICD-9	317	Mild intellectual disabilities
	318	Other specified intellectual disabilities
	319	Unspecified intellectual disabilities
ICD-10	F70	Mild intellectual disabilities
	F71	Morderate intellectual disabilities
	F72	Severe intellectual disabilities
	F73	Profound intellectual disabilities
	F78	Other intellectual disabilities
	F79	Unspecified intellectual disabilities

F. ICD-codes for Down syndrome and Neurofibromatosis

ICD-8	743.4	Neurofibromatosis
	759.30	Down syndrome
ICD-9	237H	Neurofibromatosis
	758A	Down syndrome
ICD-10	Q850	Neurofibromatosis
	Q90	Down syndrome

G. ICD-codes for congenital malformations

ICD-8	740-759.99	Congenital malformation
ICD-9	740–759	Congenital malformation
	237H	Neurofibromatosis
ICD-10	Q00-99	Congenital malformations, deformations and chromosomal abnormalities
	D82.1	Di Georges syndrom
	F84.2	Rett syndrome

Supplementary Table 4. Cancer risk for individuals with polydactyly, analyzed in regard to localization of the duplicated digit, stratified by age and sex.

	**Polydactyly**		**Thumb polydactyly**		**Finger polydactyly**	
	No. of cases polydactyly/comparisons	Adjusted* HR (95% CI)	*No. of cases polydactyly/comparisons*	Adjusted* HR (95% CI)	*No. of cases polydactyly/comparisons*	Adjusted* HR (95% CI)
**All neoplasms**	*120/5286*	1.15 (0.96-1.38)*	*19/930*	1.07 (0.68-1.70)	32/1777	0.94 (0.66-1.33)
Female	79/4159	0.96 (0.76-1.20)	14/756	0.97 (0.57-1.65)	20/1315	0.79 (0.51-1.24)
Male	41/1127	1.89 (1.38-2.58)	5/174	1.48 (0.61-3.63)	12/462	1.39 (0.78-2.48)
**Malignant neoplasms**	*55/2035*	1.40 (1.07-1.83)	8/332	1.31 (0.65-2.66)	14/780	0.92 (0.54-1.57)
**Age at diagnosis**						
0-19	*31/893*	1.80 (1.26-2.57)	<5/112	n/a	8/321	1.29 (0.64-2.60)
20 and older	*89/4393*	1.02 (0.83-1.26)	15/818	0.96 (0.58-1.61)	24/1456	0.85 (0.57-1.28)

* Adjusted for birth year, sex, county of birth, paternal age, maternal age and parental education.

Supplementary Table 5. Sensitivity analysis of cancer risk in individuals with polydactyly diagnosis at 5 years of age or younger.

	**Polydactyly**		**Isolated polydactyly**		**Polydactyly+•**		**Polydactyly+ excl. NF & DS¤**	
	No. of cases polydactyly/comparisons	Adjusted† HR (95% CI)	*No. of cases polydactyly/comparisons*	Adjusted† HR (95% CI)	*No. of cases polydactyly/comparisons*	Adjusted† HR (95% CI)	*No. of cases polydactyly/comparisons*	Adjusted† HR (95% CI)
**All neoplasms**	*107/4843*	1.13 (0.93-1.37)*	*71/3269*	1.11 (0.87-1.40)	*24/1019*	1.25 (0.84-1.88)*	21/986	1.14 (0.74-1.75)
Female	71/3775	0.96 (0.76-1.21)	49/2519	0.99 (0.75-1.32)*	13/813	0.85 (0.49-1.47)	12/786	0.81 (0.46-1.43)
Male	36/1068	1.75 (1.25-2.44)	22/750	1.50 (0.98-2.29)*	11/206	3.06 (1.65-5.67)	9/200	2.55 (1.30-5.03)
**Malignant neoplasms**	*50/1883*	1.37 (1.03-1.82)	*29/1285*	1.17 (0.81-1.69)	*18/391*	2.45 (1.52-3.95)	15/379	2.10 (1.25-3.53)
Female	*18/935*	1.00 (0.63-1.59)	*10/621*	0.85 (0.46-1.60)	*7/209*	1.76 (0.83-3.76)	6/204	1.55 (0.68-3.50)
Male	*32/948*	1.74 (1.22-2.48)	*19/664*	1.46 (0.93-2.31)	*11/182*	3.42 (1.84-6.36)	9/175	2.85 (1.44-5.62)
**Age at diagnosis**								
0-19	*29/840*	1.79 (1.23-2.59)	*15/555*	1.39 (0.83-2.32)	*12/188*	3.48 (1.93-6.28)	10/183	2.99 (1.57-5.70)
Female	*8/371*	1.12 (0.56-2.26)*	*<5/234*	n/a	*<5/89*	n/a	<5/88	2.46 (0.90-6.77)
Male	*21/469*	2.33 (1.50-3.61)*	*12/321*	1.91 (1.07-3.40)	*8/99*	4.89 (2.33-10.26)	6/95	3.78 (1.63-8.80)
20-29	*47/2247*	1.07 (0.80-1.43)	*31/1460*	1.07 (0.75-1.53)	*7/498*	0.76 (0.36-1.60)	6/484	0.66 (0.30-1.48)
Female	*37/1953*	0.97 (0.70-1.34)	*23/1264*	0.92 (0.61-1.39)	7/432	0.87 (0.41-1.83)	6/419	0.76 (0.34-1.71)
Male	*10/294*	1.73 (0.92-3.26)	*8/196*	2.05 (1.01-4.18)	*<5/66*	n/a	<5	n/a
30 and older	*31/1756*	0.89 (0.63-1.28)	*25/1254*	1.02 (0.68-1.51)	*5/333*	0.79 (0.33- 1.91)	5/319	0.83 (0.34-2.01)
Female	*26/1451*	0.90 (0.61-1.33)	23/1021	1.15 (0.76-1.75)	*<5/292*	n/a	<5/279	n/a
Male	*5/305*	0.86 (0.35-2.08)	*<5/233*	n/a	<5/41	n/a	<5/40	n/a

Presented are hazard ratios (HRs) together with 95% confidence intervals (CI) for adjusted models.
* Not meeting proportional hazards assumption
• Individuals with polydactyly and additional non-associated birth defects and/or intellectual disability
¤ Individuals in polydactyly+ cohort excluding individuals with Down Syndrome or Neurofibromatosis.
† Adjusted for sex, county of birth, paternal age, maternal age and parental education

Supplementary Table 6. Sensitivity analysis of site-specific cancer in individuals diagnosed with polydactyly at 5 years of age or younger.

	**All polydactyly**		Male		Female	
**Site-specific cancer**	*No. of cases polydactyly/comparisons*	Adjusted• HR (95% CI)	*No. of cases polydactyly/comparisons*	Adjusted† HR (95% CI)	*No. of cases polydactyly/comparisons*	Adjusted† HR (95% CI)
Leukemia	*10/256*	2.05 (1.09-3.87)	*8/151*	2.79 (1.36-5.71)	*<5*	n/a
ALL	*5/119*	2.20 (0.89-5.40)	*5/77*	3.43 (1.38-8.52)	-	-
Lymphoma	*5/202*	1.30 (0.53-3.17)	*<5*	n/a	*<5*	n/a
CNS and meningies	*9/312*	1.55 (0.80-3.01)	*<5*	n/a	*5/132*	2.02 (0.82-4.96)
Gynecological	*51/2714*	0.96 (0.73-1.26)	-	-	*51/2714*	0.96 (0.73-1.26)
Cervix	*48/2634*	0.92 (.69-1.23)	-	-	*48/2634*	0.92 (.69-1.23)
Testis	*5/190*	1.34 (0.55-3.26)	*5/190*	1.34 (0.55-3.26)	-	-
Melanoma	*8/326*	1.27 (0.63-2.56)	*<5*	n/a	*<5*	n/a
Endocrine	*6/201*	1.59 (0.70-3.60)	*<5*	n/a	*<5*	n/a

Presented are hazard ratios (HRs) together with 95% confidence intervals (CI) for both crude estimates and adjusted models. Cancer sites with 5 or more co-occuring cases are presented in the table.

* Not meeting proportional hazards assumption

• Adjusted for sex, county of birth, paternal age, maternal age and parental education

† Adjusted for county of birth, paternal age, maternal age and parental education
